# Provision of mental health services in resource-poor settings: a randomised trial comparing counselling with routine medical treatment in North Afghanistan (Mazar-e-Sharif)

**DOI:** 10.1186/1471-244X-12-14

**Published:** 2012-02-29

**Authors:** Sarah Ayoughi, Inge Missmahl, Roland Weierstall, Thomas Elbert

**Affiliations:** 1 Department of Psychology, University of Konstanz, 78457 Konstanz, Germany; 2 International Psychosocial Organisation (Ipso), 78462 Konstanz, Germany

## Abstract

**Background:**

Psychosocial stress caused by war, ongoing conflict, lack of security, and restricted access to resources promotes mental suffering and diseases in many resource-poor countries. In an exemplary setting, the present study compares the efficacy of psychosocial counselling with routine pharmacological treatment in a randomised trial in Mazar-e-Sharif (Afghanistan).

**Methods:**

Help seeking Afghan women (N = 61), who were diagnosed with mental health symptoms by local physicians either received routine medical treatment(treatment as usual) or psychosocial counselling (5-8 sessions) following a specifically developed manualised treatment protocol. Primary outcome measures were symptoms of depression and anxiety assessed before treatment and at follow-up using the Hopkins Symptom Checklist and the Mini-International Neuropsychiatric Interview. Secondary outcome measures were psychosocial stressors and coping mechanisms.

**Results:**

At 3-month follow-up, psychosocial counselling patients showed high improvements with respect to the severity of symptoms of depression and anxiety. In addition, they reported a reduction of psychosocial stressors and showed an enhancement of coping strategies. At the same time, the severity of symptoms, the quantity of psychosocial stressors and coping mechanisms did not improve in patients receiving routine medical treatment.

**Conclusion:**

These results indicate that psychosocial counselling can be an effective treatment for mental illnesses even for those living in ongoing unsafe environments.

**Trial registration:**

NCT01155687

## Background

The ongoing and escalating conflict in Afghanistan results in continuous social and sometimes traumatic stress which has an increasingly harmful impact on the mental health of the population. More than thirty years of war have left the lives of two generations of Afghans disrupted. Afghanistan is among the least developed countries in the world, ranking 181 (out of 182) nations in the Human Development Index 2009 of the United Nations.

Studies investigating the mental health status of the Afghan population report extraordinarily high levels of mental health related problems [1-4]. Lopes Cardozo and colleagues conducted a national mental health survey in 2002 and reported that 73% of the Afghans suffer from symptoms of depression, 84% from symptoms of anxiety and 59% were diagnosed with PTSD [2]. Continuous stressors, such as the loss of beloved ones, homes and jobs, poverty-related suffering, child labour, traumatising events and drug abuse affect the functioning of families and whole communities [1,5-7]. They are likely to exert their toll on the mental health of the Afghan population at large [1,5].

Therefore, the recovery of mental health and the improvement of quality of life standards constitute preconditions for building civil society and promoting peace and stability within Afghanistan [8].

However, until a few years ago, mental health facilities were practically nonexistent in Afghanistan [1,9]. A report by the World Health Organization that focused on the Afghan mental health system stated that in 2005 there were only eight psychiatrists, 18 psychiatric nurses, and 20 mental health professionals for a population of 27 million [10].

At the same time, many studies indicated that even under resource-poor and ongoing unsafe conditions with limited financial and professional resources, such as in Afghanistan, an effective treatment of mental health problems can be provided, especially through a psychotherapeutic approach [11-16]. Accordingly, Neuner and colleagues [14] showed that effective psychotherapy can be provided by persons having received no more than a short-term training of 6 weeks. The study showed that mental health of Ugandan refugees living under unstable and unsafe conditions were greatly improved. Schaal and colleagues [16], investigating counselling treatment in a war-affected sample of Rwandan genocide orphans, showed that even a small number of counselling sessions can significantly improve the mental health status of participants. These studies, showing the effectiveness of short-time interventions and the ability of counsellors with limited training to successfully conduct therapy are consistent with the findings of Rahman and colleagues [15], who trained community health workers in treating mothers with depression in rural Pakistan within four sessions. The mentioned studies indicate that counselling is feasible even with limited resources and that it is effective for populations living in conflict settings. The need of the Afghan population for mental health services was recognised by the Ministry of Public Health of Afghanistan. In a revised version of the Basic Package of Health Services (BPHS), the Mental Health Component was changed from a purely medical treatment approach (edition 2005) to a bio-psychosocial treatment approach (2010) within the BPHS [17]. As a result, psychosocial counselling services were integrated as a treatment method into the Primary Health Care System of the country. Supporting this effort and approach of the Ministry of Public Health, the Caritas Germany/EU Project provided counselling services in selected health centres affiliated with health facilities of the BPHS [17] in three provinces of Afghanistan, one being located in Mazar-e-Sharif.

To our knowledge, there have been no studies to date, which have systematically investigated the effectiveness of a psychotherapeutic intervention in Afghanistan. The present investigation was designed to fill this gap. We aimed at examining the mental health status of help seeking Afghans suffering from mental health problems. For this purpose, we assessed the severity of symptoms of depression and anxiety and the extent of psychosocial stressors. The study hypothesised that individuals who were diagnosed by local physicians with symptoms of depression and anxiety would benefit from psychosocial counselling in terms of an improvement in the primary outcome (symptom reduction). Additionally, we expected an enhancement of coping strategies and a reduction of psychosocial stressors in the counselling group. Eventually, we assumed that the change in the severity of mental health symptoms, associated with depression and anxiety, would be related (positively correlated with) to the change in the amount of social stressors reported by the patients [13].

## Methods

### Settings and local team

The present investigation was conducted in the Balkh province of Afghanistan, in its capital Mazar-e-Sharif, for the purpose of a scientific evaluation of the implementation of psychosocial counselling into the Afghan health system. The implementation process had been initialized in three provinces of the country. As the trial site, we chose Mazar-e-Sharif, 320 km northwest of Kabul, as it provided relative security and stability at the time of the study. The counselling centre was located in a suburban district.

Diagnostic interviews were conducted by two experienced local counsellors (male and female) and two international experts (both female), one fluent in the local language (Dari). A third local experienced counsellor served as a translator to one of the international experts. The international experts had received training in clinical diagnosis using structured interviews. The two experienced local counsellors had also assisted in a previous epidemiological survey in Kabul, conducted by our group [18].

Subsequent therapies were carried out at the counselling centre by newly trained local counsellors.

### Participants

In September and October 2009, a sample of 66 mental health patients (63 female, 3 male) seeking help at a Primary Health Care Centre in Mazar-e-Sharif were recruited by our team. Since the aim of our study was to assess the common treatment for mental health patients in Afghanistan, the participants were enrolled into the study solely upon the autonomous examination and subsequent referral by independent local physicians. According to their medical records none of them met our exclusion criteria (neurological disorder, mental retardation, dementia, or schizophrenia). To allow randomisation, our team allocated the participants to one of the treatment conditions based on a daily alternation routine, meaning that alternately, one day patients were allocated to the medication group, and the next day to the counselling group.

A written informed consent, explaining the procedure and the nature of the particular treatment was read out to each patient. As the illiteracy of the sample was high (73,9%), patients willing to participate gave written or oral consent. The study was approved by the Ethical Review Board of the University of Konstanz.

### Measures

All instruments were translated by local experienced counsellors into Dari using blind back translation. Discrepancies were checked by experts and a final version was derived through extensive consultation with local counsellors from the Balkh province. Due to the high illiteracy rate, all instruments were used in the form of structured interviews in which questions were read aloud to the patients.

#### Sociodemographic characteristics

Questions related to sociodemography assessed information on sex, age, ethnicity, religion, marital status, educational level, living arrangements and conditions. Additionally, we inquired into the use of medication in order to control unsupervised self-medication in both treatment groups.

##### Primary outcome measures

###### HSCL-25

The Hopkins Symptom Checklist 25 was used to screen for symptoms of depression and anxiety [19]. This screening tool is composed of a 15-item subscale for depression and a 10-item subscale for anxiety, with answer choices ranging from 1 (not at all) to 4 (extremely). It has been used widely in studies of refugees and other war-affected populations [6,20], including four studies in Afghanistan [1,2,4,5], providing outcomes at symptom, but not at diagnosis level. This screening instrument has proven to be a reliable and valid instrument for measuring symptoms of depression and anxiety in various countries and cultures [6]. Moreover, the HSCL depression scale has been found to have high reliability and validity in multiple studies with medical patients, and being sensitive to change in depressed primary care patients [19].

###### M.I.N.I

To assess whether the patients suffered from a current Major Depression, the depression section from the "Mini-International Neuropsychiatric Interview" [21] was included. The M.I.N.I. is a short structured diagnostic interview for DSM-IV and ICD-10 psychiatric disorders. Patients were asked to indicate which of the depression symptoms they had experienced within the two weeks preceding the interview. Validation studies have shown good validity and reliability in making diagnoses in less time than conventional structured interviews [21].

###### Screening for Depression

A culturally grounded assessment measure was developed in close collaboration with the "Mental Health Working Group Afghanistan", consisting of international and Afghan mental health professionals and practitioners (SAMHSA), which supports the Ministry of Public Health of Afghanistan in establishing and implementing mental health services in the country. This screening instrument explored current depressive symptoms on a 4-point-Likert-scale between 0 (never) and 3 (always). The cumulated sum of responses to the 8 items gives an image of the severity of depressive symptoms on a continuum between 0 and 24. This interview screened for culture-specific indicators of depressive symptoms and was developed to provide a first screening tool for the Primary Health Care sector in the particular Afghan context. Items concern the somatic domain (*How often do you have pain anywhere in your body that comes and goes, such as headache, stomach pain, heart racing or high blood pressure?)*, for feeling change (*How often do you experience the feeling of not caring about your family/children anymore?)*, social isolation *(How often do you feel lonely?)*, behavioural changes *(Has there been any change in your participation in everyday life?)*, harm to self or others *(How often are you so desperate or out of control, that against your will, you want to hit yourself or others?)*, suicidality *(Have you ever tried to end your life? If yes: Do you now want to end your life?) *and ability to act on one's behalf *(Have you tried out things to feel better or solve these problems?)*. Two additional questions checked for a psychosocial origin of the symptoms and drug abuse.

##### Secondary outcome measures

###### Psychosocial stressors & coping mechanisms

Interviews were conducted, according to the manual "Professional Package for Psychosocial Counsellors working in the BPHS in Afghanistan" [22], identifying psychosocial stressors and coping mechanisms among the patients. For the assessment of psychosocial stressors, a checklist of 11 different types of stressors was provided. The following psychosocial stressors, describing common problems in the Afghan society, were checked: *Family conflicts - interpersonal conflicts - difficult life transition - grief and loss - personal difficulties - sexual problems - traumatic experience - domestic violence - migration - poverty - changing gender roles and values*. Subsequently, the interviewer documented the stressors scoring each psychosocial stressor as being currently present or not present in the patient's daily life.

In order to estimate the quantity and intensity of coping strategies to stressful circumstances, an additional checklist was used. Coping mechanisms that had been reported by the patients were scored for each of the 5 items on a 4-point-Likert-skale between 0 (not at all) and 3 (fully). By this means, we explored the patients understanding *(referring to the relationship of symptoms and stressors (1)) *and manageability *(referring to the ability of creating, improving, maintaining relationships (2); solving conflicts (3) and recognizing and using own resources (4)) *of the situation as well as the value of life to them (5).

Both indicators were determined during the interview by a local psychosocial counsellor of our team.

### Intervention

#### Psychosocial counselling

Experienced local physicians, who had been trained as psychosocial counsellors in an extensive, 2-year training programme for psychosocial counselling in 2005/2006 (developed and led by IM, supported with a training on trauma treatment by TE) and gathered considerable experience in counselling there after, were educated to train Afghan women and men as psychosocial counsellors on basis of the manual "Professional Package for Psychosocial Counsellors working in the BPHS in Afghanistan" [22] approved by the Afghan Ministry of Public Health.

The 3.5-month intensive training of the 30 selected participants took place in Kabul between April and August 2009 and ended with a final examination, ensuring the required quality standard of the counsellors being set by the Ministry of Public Health. Subsequently, the newly trained psychosocial counsellors took up employment at local health care centres in three provinces in North Afghanistan, 3 of them were deployed in a counselling centre in Mazar-e-Sharif.

The counselling treatment followed the treatment guidelines of the manual "Professional Package for Psychosocial Counsellors working in the BPHS in Afghanistan" [22], which has become the official standard treatment protocol of psychosocial counsellors working in the Afghan basic healthcare system in 2009. The counselling approach has been developed and adapted to the socio-cultural background of Afghanistan by one of the authors (IM) on basis of her longstanding in-field experience. Between 2005 and 2008, approximately 11,000 patients were treated by IM and her Afghan team, consisting of experienced physicians and counsellors, in Kabul. The experiences and insights of the work with the patients were integrated into the present approach and adjusted to the specific cultural conditions. Watzlawick's short term therapy [23] and Antonowsky's salutogenetic approach [24] lie at the core of this psychosocial intervention. Additionally, selected intervention modules of Cognitive Behaviour Therapy have been included. This manual represents a resource-/and problem-solving orientated counselling approach which aims at restoring self-efficacy and developing resources, hereby enabling the Afghan patients to re-participate in their daily life in a responsible and satisfying way. Additionally, the approach is geared towards improving the patient's general physical, mental, social and spiritual health. Emphasis was put on a sense of coherence, covering comprehensibility, manageability, and meaningfulness.

The first five clearly structured counselling sessions aimed at gaining a deep understanding of the relationship between the patient's symptoms and their connection to psychosocial stressors. Following Watzlawick's ideas [23], special focus lay on the most pressing problem (main complaint) of the patient and on the identification of connections between the main complaint, current symptoms and underlying social stressors. Then, the counsellor and the patient explore possible coping mechanisms on the basis of the patient's values and resources, in order to improve the pressing psychosocial situation (self-efficacy). In case of a clinical necessity to provide the patient with further counselling, up to 8 additional counselling sessions could be added, following selected intervention modules of Cognitive Behaviour Therapy (further information is available from the authors upon request and the complete manual can be download from the official website of the Afghan Ministry of Public Health under http://moph.gov.af/en).

#### Routine medical treatment

The usual medical treatment was carried out by four local physicians, who regularly examined the patients of the control group and prescribed medication. We agreed with them on a weekly appointment and a precise documentation on the prescribed medication. This intervention can be described as the usual treatment within the Basic Public Health Care System for patients reporting mental suffering and psychosocial problems. We noted a considerable variation in prescribed medications (see Table 1).

**Table 1:** Baseline sociodemographic characteristics of patients divided by groups (*N *= 61)

	Counselling G (*N *= 31) *N *(%)	Medication G (*N *= 30) *N *(%)	p
Gender			
Women	31 (100)	30 (100)	

Ethniticity			.45
Tajik	29 (93.5)	25 (83.3)	
Pashtun	1 (3.2)	2 (6.7)	
Uzbek	1 (3.2)	3 (10)	
Religion			
Muslim	31 (100)	30 (100)	

Marial status			.69
Married	23 (74.2)	21 (70)	
Single	4 (12.9)	6 (20)	
Widowed	3 (9.7)	3 (10)	
Engaged	1 (3.2)	0 (0)	

Education			.42
None	21 (67.7)	24 (80)	
Primary school (1-4 years)	1 (3.2)	2 (6.7)	
Middle/high school (5-12)	8 (25.8)	4 (13.3)	
University	1 (3.2)	0 (0)	

Employment			.20
No	26 (83.9)	29 (96.7)	
Yes	5 (16.1)	1 (3.3)	

People living in the household			.35
1-5	1 (3.2)	5 (16.7)	
5-10	19 (61.3)	21 (70)	
10-15	10 (32.3)	3 (9.9)	
> 15	1 (3.2)	1 (3.3)	

Medication			
Pain killer	0 (0)	18 (60)	
Pail killer & sleeping pills	0 (0)	4 (13.3)	
Antidepressants	0 (0)	4 (13.3)	
Antidepress. & sleeping pills	0 (0)	3 (9.9)	
Others	0 (0)	1 (3.3)	

Age *M (range)*	31.2 (14-60)	35.3 (15-60)	.23

* = p < .01

### Procedure

The study was carried out between September 2009 and March 2010. After being randomly assigned to one of the treatment conditions, i.e. counselling or medical treatment, each patient received two initial interviews. The first one was conducted by the local experienced counsellors of our team (patients were interviewed by a counsellor of the same sex) and explored psychosocial stressors and coping mechanism. The second one was carried out by the experts from the University of Konstanz and checked for symptoms of depression and anxiety (HSCL-25; M.I.N.I.; Screening for Depression).

The routine medical treatment by the local physicians started immediately after the initial expert interview. Patients receiving medical care were treated at the local health care centre for the following 3 months. With regard to the counselling treatment group, the subsequent five manual based counselling sessions were scheduled for the following 5 weeks and carried out by the three newly trained counsellors in the counselling centre in Mazar-e-Sharif. Taking into account the specific cultural and religious setting, female participants were only counselled by female counsellors and male participants by our male counsellor. The duration of a counselling session was 45 minutes but could extend up to 60 minutes. If the newly trained counsellor had determined a respective clinical necessity, up to 8 additional counselling sessions could be added in agreement with the supervising team from Kabul In the present trial, four patients received more than 5 sessions of counselling (M = 5.16, SD = .45, ranging between 5 and 7 sessions) Three months after the first interviews, our team of experienced local counsellors from Kabul and experts from the University of Konstanz carried out a follow-up examination consisting of the previously used battery.

The interviewers who carried out the follow-up test were not fully blind to the treatment condition as the two types of intervention (psycho- vs. pharmacotherapy) were very different and thus sometimes revealed through unsolicited information given by the patient. Moreover, although the knowledge about the treatment condition was not updated before follow-up, we cannot rule out that the expert-interviewer still remembered the treatment condition of some patients.

Initial interviews took place either at the counselling centre or at the local health care centre being located next door. The follow-up interviews were carried out at the same place.

### Analysis

Descriptive data are expressed as frequencies (%), mean scores, and standard deviations. Baseline characteristics of the groups were compared using chi-squared tests and Fisher's exact tests to examine the effects of randomization. Between-group differences at pre-test and follow-up were analysed using independent-sample t tests, Mann-Whitney U tests and analysis of covariance.

For the outcome variables we calculated repeated-measures analyses of variance (ANOVA) with time of assessment (pre-test and 3-month follow-up) as the within-subject factor and treatment condition (counselling and medication) as the between-subject factor. For significant results, changes within the particular treatment group from pre-test to follow-up were analysed using binominal tests and sample t tests. Within-treatment effect sizes (Cohen's d) were computed for both treatment conditions. To control for mediation effects we carried out linear forced entry regression analyses. The assumption of homogeneity of variance was tested using the Levene test at a significance level of p > .05. Kolmogrov-Smirnov tests were used to determine normal distribution. Data analysis was performed using PAWS Statistics 18.0 [25] and R for Mac OSX Version 2.11.1 (R Development Core Team 2010).

## Results

### Baseline characteristics

Table 1 gives a demographic overview of the sample, separately for each treatment condition. There were no systematic group differences in any of the socio-demographic variables. Patients of the counselling group reported no use of medication (herbal medication included) during the treatment or follow-up period and patients of the medication group (routine medical treatment) did not take any non-prescribed medication. For a detailed description of the prescribed medication see Table 1.

### Dropouts

**Figure 1:**
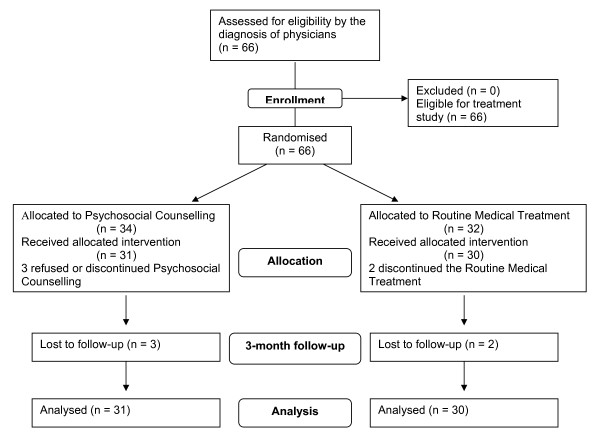
**Flowchart of the study protocol**.

In the counselling group, 3 patients (8.8%), one being male, dropped out of treatment. In the medication group (routine medical treatment), 2 patients (6.3%), both being male, did not complete treatment (see flow chart in Figure 1). Men dropouts of both treatment conditions reported not being able to afford the time to continuously visit the distantly located health **care centre**.

### Symptom reduction

**Figure 2:**
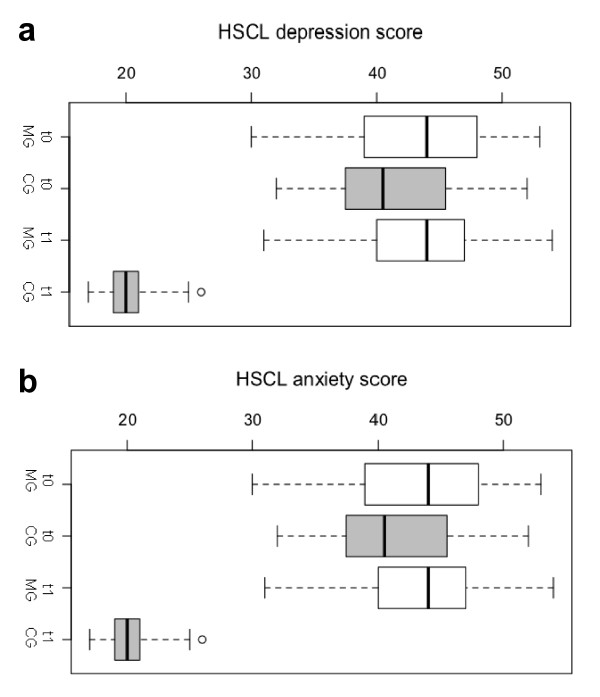
**Change in HSCL-Depression/-Anxiety scores. a. **Change in HSCL-Depression scores. **b.** Change in HSCL-Anxiety scores.

We found a significant interaction of treatment × time in the HSCL depression score (*F*(1,59) = 175.21, *p *< .001, *η*_p_^2 ^= .75; which also produced a main effect (*F*(1,59) = 142.60, *p *< .001, *η*_p_^2 ^= .71). As illustrated in Figure 2a, pre-treatment depression scores were the same for the two groups (counselling *M *= 41.65, *SD *= 6.03 and medication group *M *= 43.00, *SD *= 6.53; two sample t-test, *t*(60) = -.84, *p *= .40, *d *= .22). At 3-month follow-up, the psychosocial counselling group showed significantly lower HSCL depression scores (*M *= 20.26, *SD *= 1.95) than the medication group (*M *= 44.10, *SD *= 5.64) (Mann-Whitney *U *test, *U *= .00, *z *= -6.73, *p *< .001, *r *= 0.86). While we found a large treatment effect size for the psychosocial treatment group in the reduction of the HSCL depression score (*M *= -21.39, *SD *= 6.54, one sample *t*-test, *t*(30) = -18.21 *p *< .001, *d *= 3.27), the change in the HSCL depression score in the medication group indicated that there was no improvement (*M *= 1.10, *SD *= 6.73, one sample t-test, *t*(29) = .90, *p *= .38, *d *= .163). A last observation carried forward analyses considering participants that dropped out revealed a similarly large significant treatment effect (Mann-Whitney *U *test, *U *= 16.50, *z *= -6.79, *p *< .001, *r *= 0.84).

Similar results were obtained for the anxiety scores (Figure 2b). In a repeated measures analysis of variance (ANOVA) with the HSCL anxiety score at pre-treatment and follow-up as the within-subject factor and psychosocial counselling versus medication as between-subject factor was calculated. Again, a significant interaction indicated that the HSCL anxiety score decreased in the counselling but not the medication group (*F*(1,59) = 172.46, *p *< .001, *η*_p_^2 ^= .75; main effect *F*(1,59) = 198.89, *p *< .001, *η*_p_^2 ^= .77). Both groups had nearly identical values in their HSCL anxiety scores at pre-treatment (psychosocial counselling group: *M *= 29.52, *SD *= 4.63, medication group: *M *= 30.63, *SD *= 4.22, two sample *t*-test, *t*(60) = -.983, *p *= .329, *d *= .25). At the time of post-treatment, the counselling group showed lower HSCL anxiety scores (*M *= 12.68, *SD *= 1.33) than the medication group (*M *= 30.03, *SD *= 5.13) (Mann-Whitney U test, *U *= 1.00, *z *= -6.74, *p *< .001, *r *= 0.86). A last observation carried forward analyses revealed the treatment effect were about the same when drop outs are considered in the calculation (Mann-Whitney U test, *U *= 19.00, *z *= -6.78, *p *< .001, *r *= 0.83). Again, the reduction of the HSCL anxiety scores revealed a large treatment effect size in the psychosocial counselling group (*M *= -16.84, *SD *= 4.87, one sample *t*-test, *t*(30) = -19.24, *p *< .001, *d *= 3.46), while the change in the medication group was negligible (*M *= -.60, *SD *= 4.78, one sample t-test, *t*(29) = -.69, *p *= .497, *d *= .125). Thus, only the psychosocial counselling significantly improved the depression and anxiety symptoms.

These findings are validated by further assessments through the M.I.N.I. and the Screening for Depression. Pre-treatment diagnoses of current major depression assessed through the M.I.N.I. did not differ between both groups (counselling group *N *= 27 (87.1%), and medication group *N *= 27 (90%); *χ^2 ^*(1) = .17, *p *= 1.00). The percentage of counselling treatment patients meeting M.I.N.I. criteria for a diagnosis of a current major depression dropped to 0%, whereas 28 (93,3%) patients of the medical treatment met M.I.N.I. criteria for such a diagnosis at follow-up. The counselling and medication group significantly differed in the status of diagnosis for current major depression at follow-up (*χ^2 ^*(1) = 56.12, *p *< .001).

The results of the Screening for Depression also show that in the counselling group the depression score significantly changed between pre-test and follow-up (*M *= -10.97, *SD *= 2.87, one sample t-test, *t*(30) = -46.97 *p *< .001, *d *= 3.82). At the same time, the medication group showed an increase in symptom severity (*M *= 1.50, *SD *= 2.87, one sample t-test, *t*(29) = 2.81, *p *< .01, *d *= .57). Additionally the Screening for Depression shows high correlations with the MINI *(r *= .424**) and the depression section of the HSCL 25 (*r *= .682**).

### Psychosocial stressors

Besides depression and anxiety symptoms, we assessed the psychosocial stressors reported by the patients. The average number of reported current psychosocial stressors was 3.51 (*SD *= 1.41) for the whole sample. There was no significant difference between the groups before the treatment (psychosocial counselling group: *M *= 3.29, *SD *= 1.37, medication group: *M *= 3.20, *SD *= .96, two sample t-test, *t*(60) = -.30 *p *= .768, *d *= .08).

**Figure 3:**
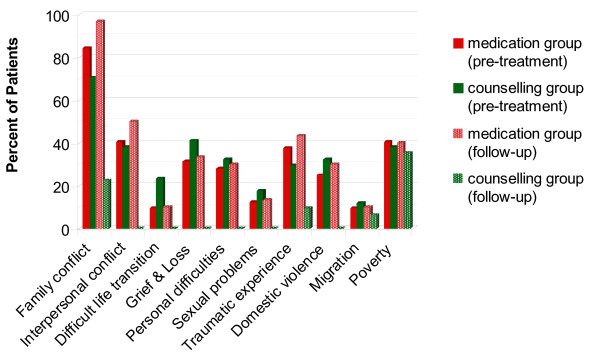
**Psychosocial stressors**.

The most frequent psychosocial stressor types were, family conflicts (*n *= 47; 77%) and (inter)personal problems and difficulties such as issues of honour and shame (*n *= 41; 67.2%). In addition, ongoing domestic violence appeared to be not unusual among interviewed patients (*n *= 16; 26.2%). After the treatment, the patients in the counselling group reported fewer psychosocial stressors (*M *= .74, *SD *= .68) than the patients in the medication group (*M *= 3.57, *SD *= 1.01) (Mann-Whitney *U *test, *U *= 8.00, *z *= -6.71, *p *< .001, *r *= .86). Apart from poverty (35.5%), all reported stressors dropped to under 5% in the counselling group. Figure 3 provides a more detailed look at the specific types of psychosocial stressors and specific changes within treatment time for both groups.

Counselling resulted in a large effect size for the reduction in the psychosocial stressors (*M *= -2.55, *SD *= 1.18, one sample *t*-test, *t*(30) = -12.04, *p *< .001, *d *= 1.91), whereas the patients of the medication group felt that their psychosocial stressors occurred more frequently at follow-up (*M *= .37, *SD *= .61, one sample *t*-test, *t*(29) = 3.23, *p *= .003, *d *= .61).

#### The effect of psychosocial stressors on the symptom reduction

We found a high correlation between the reduction of psychosocial stressors and the symptoms for both HSCL scores, depression (*r_p _*= .81, *p *< .001) and anxiety (*r_p _*= .82, *p *< .001). To analyse whether there was a treatment effect on the HSCL depression change in the counselling group besides the one reduction in psychosocial stressors, we performed a mediation analyses for the mediator *psychosocial stressors*. For the depression symptoms, the change in the HSCL depression score between pre-treatment and follow-up (*HSCL depression change*) was regressed on the change in psychosocial stressors (*psychosocial stressors change*) as well as on the *treatment *that was dummy coded with "0" for the medication group and "1" for the psychosocial counselling group in a first step. In a second step, the *psychosocial stressor change *was regressed on *treatment*.

**Figure 4:**
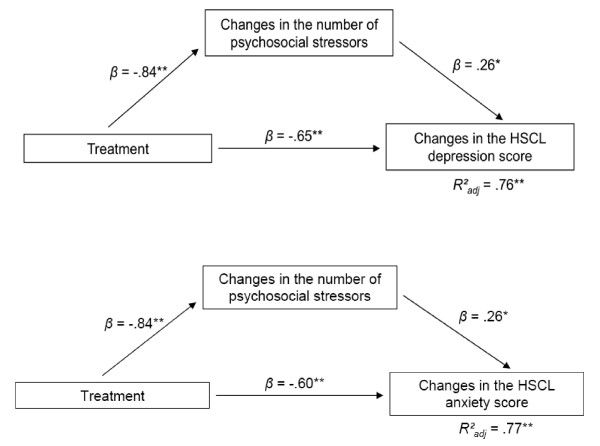
**The effect of psychosocial stressors on the treatment outcome regarding depression and anxiety. a. **The effect of psychosocial stressors on the treatment outcome regarding depression. **b.** The effect of psychosocial stressors on the treatment outcome regarding anxiety.

Figure 4a shows that the reduction in the number and frequency of psychosocial stressors contributes to the reduction in the HSCL depression score (*β*_psychosocial stressor change _= .26, *p *= .031) as indicated by the high correlation between these two variables. Still, as the treatment also accounts for the reduction in psychosocial stressors (*β*_treatment _= .-84, *p *< .001), the treatment outcome in the HSCL depression score changes is therefore mediated via this indirect treatment effect. Moreover, there is also a direct treatment effect beyond the reduction of psychosocial stressors accounting for changes in the HSCL depression score (*β*_treatment _= .-65 *p *< .001). These two variables are responsible for 76% of the variance in the HSCL depression score changes (*R*_adj_^2 ^= .76, *F*(2,58) = 95.87, *p *< .001, *f*^2 ^= 3.17). The sample size for the regression analysis was sufficient as indicated by the calculated power ((1 - *β) *= 1.00) using G × Power 3 [26]. Moreover, a further analysis of the residuals on collinearity, normal distribution of the residuals, homoscedasticity and influence on outliers indicated that the proposed model fulfils all necessary quality criteria. Additionally, the interaction term treatment × psychosocial stressor change did not explain more variance significantly *F*(1,57) = .31, *p *= .579) indicating that the psychosocial stressor change mediates but not moderates the treatment effect. Taken together, the psychosocial counselling had a large effect on the HSCL depression score changes.

The same analysis was calculated for changes in the HSCL anxiety score (Figure 4b). The sample size was sufficiently large for the regression analyses ((1 - *β) *= 1.00).

The reduction in the psychosocial stressors contributed to the reduction in the HSCL anxiety score (*β*_psychosocial stressor change _= .26, *p *= .031), likewise to the HSCL depression score. Also, the treatment accounted for the reduction in psychosocial stressors (*β*_treatment _= .-84, *p *< .001) and consequently had an indirect effect on the treatment outcome via this path. Moreover, also a direct treatment effect accounted for changes in the HSCL depression score (*β*_treatment _= -.60, *p *< .001). The regression model that included the two variables, treatment and psychosocial stressor change, had a large effect on the variance of the HSCL anxiety score (*R*_adj_^2 ^= .77, *F*(2,58) = 98.51, *p *< .001, *f*^2 ^= 3.35). The power in this model was sufficient ((1 - *β *= 1.00). As for the preceding model on HSCL depression change, an analysis of the residuals revealed that all necessary quality criteria were met. Moreover, there was no moderation effect of the psychosocial stressors.

### Coping mechanism

Besides psychosocial stressors, we assessed coping mechanisms of the whole sample, with 56 (91.8%) patients being unable (scoring 0 and 1 on a 4-point likert-scale) to recognize a relationship between own symptoms and specific psychosocial stressors before treatment. At the same time, 55 (90.2%) patients were not able to manage or solve current conflicts (scoring 0 and 1 on a 4-point likert-scale). Again, at follow-up there was a significant between-group difference (*t*(59) = -28.58; *p *< .01, with counselling patients showing improved coping strategies in all categories at follow-up (*M *= 2.39; *SD *= .34; *t*(30) = 28.33; *p *< 0.01). Such a change did not occur in the medication group (routine medical treatment) (*M *= .33; *SD *= .21; *t*(29) = -8.43; *p *< .01).

## Discussion

We carried out a randomised clinical trial of psychosocial counselling in a sample of help-seeking Afghans suffering from psychosocial stress and mental illnesses. We assessed depression and anxiety symptoms, psychosocial stressors and coping abilities at pre-treatment and at 3-month follow-up to measure the stability of the treatment effects.

We found high rates of symptoms of depression and anxiety in the help-seeking patients prior to treatment. These findings are in line with surveys assessing prevalence rates of mental health symptoms among the Afghan population [1-4]. According to the interviews with the patients, factors that may have contributed to the high rates of depression and anxiety include daily stressors, such as loss of family members, ongoing war and poverty as well as continuous feelings of hopelessness and helplessness. These results are in line with findings of Miller and colleagues [5] in Afghanistan.

The assumption that the counselling treatment would be superior to the routine medical treatment in terms of reducing symptoms and psychosocial stressors at follow-up was supported. Counselled patients reported fewer symptoms of anxiety and depression and less psychosocial stressors after the therapy, whereas these symptoms and stressors remained stable in the pharmacological treatment group. Actually, patients who had received routine medical treatment but no counselling, reported an increase of social stressors at the follow-up interview, indicating that prescription of medication had neither improved daily living conditions and nor coping strategies. This lack of significant effects in the medication condition could relate to the suboptimal pharmacological treatment. However, we would like to emphasize that, in line with Fournier's findings, the magnitude of benefit of antidepressant medication compared with placebo may be minimal or nonexistent, on average, in patients with mild to moderate depression symptoms [27]. Thus, the current effects may not necessarily be much improved with an optimised psychopharmacological treatment in this group of help-seeking patients.

Measures assessing symptom-reduction included the M.I.N.I., which revealed significant correlations with the depression scale of the HSCL 25. However, interferences from the HSCL 25 pertaining to depression and anxiety as psychiatric disorders have to be drawn with caution because of cultural specifics as already pointed out by Ventevogel and colleagues [9] in an earlier study in Afghanistan.

Arguably, the possibility to address ongoing stressful living conditions in the counselling sessions, taken by itself, considerably contributed to the massive treatment effects. Nevertheless, the counselling treatment enabled patients to gain a variety of coping abilities to successfully face the ongoing stressful living conditions in Afghanistan and to regain influence over their lives, thereby reducing the extent of social stress which is known to aggravate or even cause mental health problems [1,5].

The improvement in mental health status following counselling found in the present investigation is consistent with the results of previous studies [11-16]. In line with this work, the present study showed that local men and women can be trained in a reasonable time period to effectively treat patients suffering from mental health problems. Additionally, the present findings indicate that a mere five counselling sessions can already improve the patient's mental health status significantly, corresponding with findings of Rahman [15] and Schaal et al. [16].

This indicates that psychosocial care can be an effective treatment even in low-resource settings of a war-affected country like Afghanistan.

Limitations of the study include that the number of counsellors and doctors involved was limited and the assessment was restricted to only one site. Security issues made it difficult to include more sites. Therefore, any generalization must be considered with care. A further methological limitation is that the interviewers were not fully blind as to the treatment condition. For logistical and security reasons, our team was only able to send one Dari-speaking expert to Mazar-e-Sharif. This expert was commissioned to supervise the execution of the study, but was also involved in the conduction of interviews.

Finally, long-term effects should be investigated in order to potentially improve therapeutic techniques of the counselling treatment. As the counselling approach is a combination of different modules, hence a dismantling would be useful in further studies.

## Conclusion

To our knowledge, we have conducted the first randomised controlled trial of a counselling treatment in a sample of mental health patients in Afghanistan. In sum, we demonstrated that culturally sensitive counselling which draws on the personal resources of the patients and targets present psychosocial stressors can be an effective treatment in this war-affected society and under unsafe living conditions. The limited need of resources suggests that counselling service in countries comparable with Afghanistan may have the potential to support the re-establishment of well-functioning families and whole communities.

## Competing interests

The authors declare that they have no competing interests.

## Authors' contributions

SA designed and carried out the treatment studies, analysed the results and prepared the manuscript. IM participated in the training of the local Counsellors, designing and coordinating the studies and provided professional feedback in interpreting the results. RW participated in the statistical analyses of the results. TE participated in designing the study and provided scientific feedback and support in interpreting the findings and drafting the manuscript. All authors read and approved the final manuscript.

## Pre-publication history

The pre-publication history for this paper can be accessed here:

http://www.biomedcentral.com/1471-244X/12/14/prepub
